# Relationship between Remote Sensing Data, Plant Biomass and Soil Nitrogen Dynamics in Intensively Managed Grasslands under Controlled Conditions

**DOI:** 10.3390/s17071483

**Published:** 2017-06-23

**Authors:** Christoph Knoblauch, Conor Watson, Clara Berendonk, Rolf Becker, Nicole Wrage-Mönnig, Florian Wichern

**Affiliations:** 1 Faculty of Life Sciences, Rhine-Waal University of Applied Sciences, Marie-Curie-Str. 1, 47533 Kleve, Germany; conor.watson@hsrw.eu (C.W.); florian.wichern@hsrw.eu (F.W.); 2 Versuchs- und Bildungszentrum Landwirtschaft Haus Riswick, Elsenpaß 5, 47533 Kleve, Germany; clara.berendonk@lwk.nrw.de; 3 Faculty of Communication and Environment, Rhine-Waal University of Applied Sciences, Friedrich-Heinrich-Allee 25, 47475 Kamp-Lintfort, Germany; rolf.becker@hochschule-rhein-waal.de; 4 Grassland and Fodder Sciences, Faculty of Agriculture and the Environment, University of Rostock, Justus-von-Liebig-Weg 6, 18059 Rostock, Germany; nicole.wrage-moennig@uni-rostock.de

**Keywords:** precision agriculture, fertilizer response, vegetation indices, nitrous oxide emission, *Lolium perenne*, sensor network

## Abstract

The sustainable use of grasslands in intensive farming systems aims to optimize nitrogen (N) inputs to increase crop yields and decrease harmful losses to the environment at the same time. To achieve this, simple optical sensors may provide a non-destructive, time- and cost-effective tool for estimating plant biomass in the field, considering spatial and temporal variability. However, the plant growth and related N uptake is affected by the available N in the soil, and therefore, N mineralization and N losses. These soil N dynamics and N losses are affected by the N input and environmental conditions, and cannot easily be determined non-destructively. Therefore, the question arises: whether a relationship can be depicted between N fertilizer levels, plant biomass and N dynamics as indicated by nitrous oxide (N_2_O) losses and inorganic N levels. We conducted a standardized greenhouse experiment to explore the potential of spectral measurements for analyzing yield response, N mineralization and N_2_O emissions in a permanent grassland. Ryegrass was subjected to four mineral fertilizer input levels over 100 days (four harvests) under controlled environmental conditions. The soil temperature and moisture content were automatically monitored, and the emission rates of N_2_O and carbon dioxide (CO_2_) were detected frequently. Spectral measurements of the swards were performed directly before harvesting. The normalized difference vegetation index (NDVI) and simple ratio (SR) were moderately correlated with an increasing biomass as affected by fertilization level. Furthermore, we found a non-linear response of increasing N_2_O emissions to elevated fertilizer levels. Moreover, inorganic N and extractable organic N levels at the end of the experiment tended to increase with the increasing N fertilizer addition. However, microbial biomass C and CO_2_ efflux showed no significant differences among fertilizer treatments, reflecting no substantial changes in the soil biological pool size and the extent of the C mineralization. Neither the NDVI nor SR, nor the plant biomass, were related to cumulative N_2_O emissions or inorganic N at harvesting. Our results verify the usefulness of optical sensors for biomass detection, and show the difficulty in linking spectral measurements of plant traits to N processes in the soil, despite that the latter affects the former.

## 1. Introduction

In the context of the recent changes of the European milk market with the termination of the market quota regulation, dairy farmers will have to improve the efficiency of their enterprises [1]. At the same time, the European Common Agricultural Policy and the European Water Framework Directive require a substantial reduction of the negative effects of farming [2]. Consequently, dairy farms have to become both more environmentally friendly and more profitable.

For profitability, dairy farmers often try to improve the fodder production by intensifying their meadow use by harvesting repeatedly, accompanied by intensive fertilization and ryegrass (*Lolium perenne* L.) dominated swards. Nitrogen (N) input is the centerpiece of this intensification, as it increases the chlorophyll (and protein) content and plant biomass. However, as the fertilizer N input increases, N losses, such as gaseous emissions of nitrous oxide (N_2_O) or ammonia (NH_3_), and nitrate (NO_3_^−^) leaching, also increase [3]. In addition to fertilizer, N mineralized from soil organic matter is an important source of plant nutrition in grassland systems. The microbial biomass is the main driver of this process, and at the same time it provides an important intermediate N sink, exceeding those of inorganic soil N or plant N in the harvested biomass [4]. The findings on the effects of N fertilization on mineralization processes in grassland soils are, to some extent, contradictory [5], but in general, they show that low-mineral N fertilization increases mineralization, whereas high N levels decrease it. On the other hand, enhanced N levels in soil have not been shown to affect the soil microbial biomass [6]. Consequently, for improving the N-use efficiency of grasslands, available N has to be assessed, and N mineralization has to be considered.

Therefore, one aim of more sustainable dairy farming systems should be to increase N-use efficiency and reduce N losses, especially in intensively managed grassland systems. To this end, farmers have to better understand and be able to predict the grass’ biomass development, N demand, and N availability throughout the growing season, allowing for adjustments of fertilizer application in relation to the biomass development. One possibility to quickly estimate the plant biomass in the field is the use of remote sensing techniques, such as optical sensors based on measuring light reflectance from vegetation in the range of red to near-infrared light (approximately 630–900 nm), or hyperspectral sensors [7,8,9]. Ideally, these remote sensing data will, in future, feed into a predictive model integrating plant biomass development, plant N uptake, and N delivery from N mineralization. Such a model would allow farmers to adjust their N fertilizer application rates after each grass harvest in relation to plant demand, and would provide valuable information on N losses and thus support higher N-use efficiency. To develop the model, basic relationships between N fertilization level, reflection patterns, plant biomass, and soil N dynamics have to be established. It has been shown that plant biomass and biochemical traits of above-ground biomass can be predicted using hyperspectral sensors and vegetation indices based on a range of spectral bands [7], but also by more simple sensors and vegetation indices, such as the NDVI (normalized difference vegetation index) [8,10]. Plant N uptake is affected by the available N in the soil, and therefore, N mineralization has to be considered. To our knowledge, this cannot be easily determined indirectly. However, recently, hyperspectral reflectance data of plant canopies has been used to detect differences in soil microbial community compositions in artificial soil systems [11]. Nevertheless, no mechanistic explanation was given, thus leaving unexplained correlations. If plant canopy reflectance data and vegetation indices such as the NDVI can be used to differentiate between soil microbial communities, we can assume that N mineralization as one major function of the soil microbial biomass might also be related to the spectral reflectance of the plant canopy. Another important pathway of N losses in intensively fertilized grassland that has to be considered is the emission of N_2_O [12,13]. In research studies, N_2_O emissions can be assessed in the field using, for example, photoacoustic infrared or laser sensors, or by gas sampling and subsequent gas chromatography, which, however, is more laborious and requires sophisticated field and lab equipment. As N_2_O emissions are related to the available N in the soil, we can assume a relationship with the N fertilization level.

Under field conditions, a strong spatial variability of reflectance values, as affected by soil heterogeneity and temporal variability due to changing nutrient and water availabilities, is common [7,14]. Consequently, to predict the relationship between the fertilizer level, grassland biomass and associated N fluxes using remote sensing data—which is one approach, among others such as machine-learning algorithms—as a basis for model developments, experiments under controlled conditions are required. Besides reducing spatial variability, temporal changes in the air temperature and soil moisture can be limited, allowing a focus on the main N effects.

Against the background of these considerations, we propose: (1) that elevated N fertilizer levels increase plant biomass and gaseous N losses; (2) that optical sensors can be used to detect plant biomass changes; and (3) that a close relationship between the N level, plant biomass and N losses exists. In a pot experiment under controlled conditions, we therefore investigated the relationship between the N fertilizer level, plant biomass of permanent *Lolium perenne* L. swards, and N dynamics as reflected by N_2_O emissions and inorganic N levels at the end of the experiment, with the canopy reflectance using an optical sensor. The objectives were to: (1) evaluate the relationship between the growth response of permanent grasslands to increasing N levels (under controlled conditions, where some of the field variability was excluded) and vegetation indices as measured with optical sensors; (2) estimate the fertilization-associated N_2_O losses using a photoacoustic sensor; (3) measure effects on the soil microbial biomass as the driver of mineralization in soils; and (4) evaluate the relationship between vegetation indices and N_2_O losses or inorganic N.

## 2. Materials and Methods

### 2.1. Experimental Design and Soil Sampling

A pot experiment was performed in 2014 at the greenhouse of the Rhine-Waal University of Applied Sciences in Kleve, Germany. The set-up and experimental procedure were chosen to imitate the conditions and characteristics of a fertilization field trial in an intensified permanent grassland system at the Lower Rhine region (51°46′54′′ N, 06°09′47′′ E; 13 m a.s.l.), an important dairy farming area with a permanent grassland and a moderate temperate climate, allowing intensive grassland use. To this end, undisturbed *Lolium perenne* L. sods (0 to 10 cm) were taken from the control plot of the field trial. Furthermore, soil from the A_h_ horizon of the same plot (up to a 30 cm depth) was collected as an additional substrate for the pot experiment, sieved (<4 mm), and mixed thoroughly for homogeneity. The gravimetric water content (105 °C; 48 h) and water holding capacity [15] were measured. The examined soil was classified as a gleyic cambisol [16] with a silty clay texture (414 g·kg^−1^ clay/524 g·kg^−1^ silt) in the A_h_ horizon. The basic soil properties were: pH 6.1 (1:5 soil/water), extractable P of 74 mg·kg^−1^ (CAL—calcium acetate lactate; 1:10 soil/extract ratio), extractable K of 427 mg·kg^−1^, and extractable Mg of 300 mg·kg^−1^.

Grass sods and prepared soil were reassembled in 12 L polyvinyl chloride pots (surface area: 380 cm^2^) and placed in a programmable plant growth chamber. The environmental conditions were kept at a similar level throughout the 100 days of the experiment (day-to-night ratio: 2/1; irradiance: not less than 75 W·m^−2^; daytime temperature: 20 °C; and night-time temperature: 15 °C). To ensure ideal and standardized conditions, all pots were adjusted to a 50% water holding capacity by rewetting the soil manually. Furthermore, a wireless sensor cluster network was established, monitoring the soil temperature and volumetric water content. Each pot contained a single sensor (Spade, Sceme) at a 10 cm depth with a temporal measuring resolution of 15 min. The soil water content was measured by time domain reflectometry [17].

Two weeks later, *Lolium perenne* swards were cut to 5 cm heights, initializing the start of the experiment. Four N fertilizer treatments were established: 0 (control treatment), 85 N, 170 N and 340 N, with the numbers representing the total amount in kg·N·ha^−1^ of the mineral fertilizer calcium ammonium nitrate (CAN; 26% N). CAN was applied in four portions, where the first application was carried out directly after the pre-cut. The remaining three fertilizations were done at the end of each growth period, after harvesting (days 24, 49 and 73). The fourth and last harvest event took place on day 100. Grass plants were cut with scissors to 0.05 m heights. Afterwards, the obtained biomass was dried for 2 days at 60 °C to calculate the dry matter yield. The pot experiment was set up as a completely randomized block design with seven replicates per treatment (28 pots).

### 2.2. Gas Flux Measurements

During the pot experiments, gas flux rates of CO_2_ and N_2_O for each pot were detected on a regular basis. In total, 31 measurements were carried out throughout the 100 days of the experiment. Flux rates were measured using a closed dynamic chamber system [18,19]. Therefore, a photoacoustic field gas-monitor (1412) linked with a multipoint sampler (1309; both devices: INNOVA, LumaSense Technologies A/S, Ballerup, Denmark) was deployed to detect CO_2_ and N_2_O concentrations. Briefly, chambers were placed on collars for intervals of 25 to 30 min. Each collar was pressed 0.05 m into the soil to ensure the detection of soil–air exchange fluxes for a defined area. The collars were left in the pot throughout the experiment. The cylindrical chambers used were made of PVC with a volume of 7.98 L. The time to complete one measurement was 66 s, with a sample integration time of 5 s per gas and water vapor. Six chambers were analyzed alternately via a multipoint sampler, so that five measurements for each pot were feasible within 30 min. Chambers and measuring devices were connected by Teflon tubes with an inner diameter of 3 mm. All tubes had the identical length of 5 m. The flushing time of the tubes before each measurement was adjusted to 3 s, with 8 s for the chambers. To minimize side effects, auto-compensation for water vapor interference and cross interference was activated. No fan was used in the chamber. Chamber fluxes were calculated by regression analyses, fitting a linear function to the time series of the two gases. To avoid abrupt changes in the soil moisture and temperature, gas measurements were conducted at noon.

### 2.3. Narrow-Band Vegetation Indices

The optical sensor GreenSeeker (NTech Industries, Inc., Ukiah, CA, USA) was used in the experiment. The GreenSeeker provides measurements for two wavelengths (650 ± 10 nm and 770 ± 15 nm), enabling the calculation of the normalized difference vegetation index (NDVI) and the simple ratio (SR) (Table 1).

Spectral measurements by the sensor were carried out before harvest events and concurrently with gas flux measurements. For each pot, the mean value of three independent measurements was calculated. The distance between the plant surface and GreenSeeker was 30 cm at the nadir position. The light conditions were kept constant, as artificial light was switched on. The influence of varying water conditions on the leaves was minimized by irrigation, taking place not before, but after the spectral measurements.

### 2.4. Soil Analysis

At the end of the greenhouse experiment, a soil aliquot of each pot was used to analyze inorganic N, total organic C and total N, and extractable C and N, as well as soil microbial biomass C (MBC) and soil microbial N (MBN).

MBC and MBN were analyzed by chloroform fumigation-extraction [22,23]. A homogeneous soil sample was divided into portions for fumigation- or non-fumigated-extraction. In detail, 10 g of soil (on an oven-dry basis) was fumigated inside a vacuum desiccator at 25 °C with ethanol-free chloroform (CHCl_3_). After 24 h, the chloroform was removed with a 6-fold evacuation of the desiccator. The soil samples were then extracted with 0.5 M K_2_SO_4_, and placed on a horizontal shaker at 200 rpm for 30 min. After shaking, all extracts were filtered (VWR 305; particle retention: 2–3 µm). Non-fumigated samples were extracted in the same way as fumigated samples. Extracts were then stored until further analysis at −18 °C to avoid microbial transformations and nitrification processes. Prior to the analyses, all extracts were rapidly thawed to room temperature. Extractable C and N in all K_2_SO_4_ extracts were detected after combustion at 800 °C using a Multi N/C 2100S analyzer (Analytic Jena, Jena, Germany). MBC was calculated as the ratio of extractable C (*E*_C_) and *k*_EC_, with *E*_C_ being the difference in organic C extracted from fumigated and non-fumigated samples. The coefficient *k*_EC_ represented the fraction of microbial C released in 24 h of fumigation. Its value for the calculation was 0.45 [24]. MBN was calculated as *E*_N_/*k*_EN_, with *E*_N_ being the difference in organic N extracted from fumigated samples and non-fumigated samples. The coefficient *k*_EN_ represented the fraction of microbial N, with *k*_EN_ = 0.54 [22,25]. Extractable organic C (EOC) was defined as the 0.5 M K_2_SO_4_-extractable organic C from the non-fumigated sample. Likewise, extractable organic N (EON) was defined as the difference between the total extractable N and inorganic N (NO_3_ + NO_2_ + NH_4_) after extraction with 0.5 M K_2_SO_4_.

The organic C and total N of the original soil were determined gas-chromatographically via dry combustion at 1020 °C using an elemental analyzer (Carlo Erba NA 1500). Subsamples were dried before detection for 24 h at 105 °C, and homogenized in a ball mill for 2 min at a frequency of 25 rev s^−1^. Inorganic N was measured in the extract of the non-fumigated samples. Data for nitrate, nitrite and ammonium were obtained within an aliquot of each extract using a continuous segmented flow analyzer (AutoAnalyzer 3 HR, Seal Analytical, Norderstedt, Germany).

### 2.5. Statistical Analysis

Data were analyzed by a one-way analysis of variance, followed by a post hoc Tukey’s HSD (honest significance difference) test to identify treatment differences. As a prerequisite for the analysis, data were tested for normality (Shapiro-Wilk test) and for homogeneity of variances (Levene’s test). All statistical procedures were done using R software (version 3.0.1, R Development Core Team, 2012). Figures were generated using MATLAB (MathWorks, version 9.0, R2016a).

## 3. Results

### 3.1. Temporal Dynamics of Soil Moisture and Temperature

**Figure 1:**
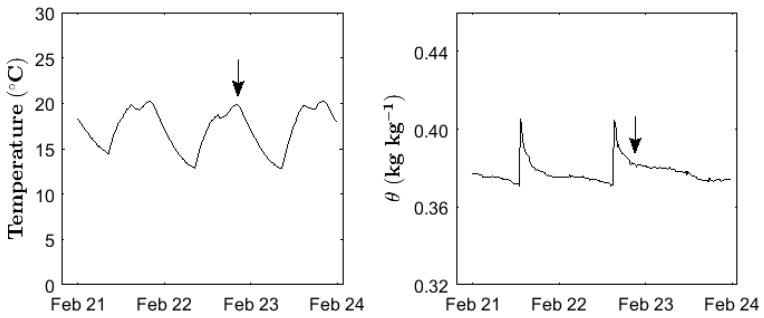
A typical time series of soil temperature (**left**) and gravimetric soil water content *θ* (**right**) of a sample pot during the 100 days of the greenhouse experiment. Arrows indicate a measurement event of greenhouse gas emissions at a temperature plateau, and after the sharp and sudden increase and decrease of *θ* caused by irrigation.

The soil temperature during the 100 days at a depth of 10 cm varied between 12.2 °C and 25.3 °C, with an average daily temperature of 17.8 °C. From the end of March (day 85), higher irradiance by the increasing day-length and sunlight was observed, causing an upward shift of the temperature profile by 1.8 °C. Before that, the temperature profile showed a constant daily variation around a mean temperature value of 17.4 °C. In general, minimum temperatures during the day were reached at 7 am, followed by a sharp increase over the next 3 h. Until evening, the temperature level remained on a plateau of 20 °C, and slowly decreased during the night. The soil moisture was mainly influenced by irrigation, which caused an increase in the gravimetric water content by 5%, and not by irradiance (Figure 1).

### 3.2. Plant Biomass Development

**Figure 2:**
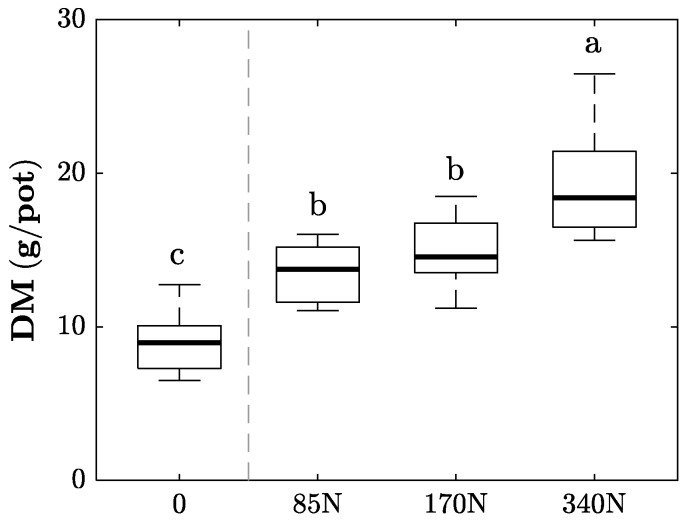
Cumulative above-ground plant dry matter of *Lolium perenne* L. at the end of the experiment after four cuts carried out over 100 days. Different letters indicate significant differences at *p* < 0.05.

Above-ground dry matter of *Lolium perenne* increased until each harvest event, and was always higher for the fertilized treatments (data not shown). Cumulative dry matter at the end of the experiment was significantly larger for the fertilized treatments compared to the control (Figure 2). Compared to the control, the 85 N treatments had on average a 49% larger yield, and the 170 N and 340 N treatments 64% and 113% larger yields, respectively. However, cumulative above-ground biomass of treatments 85 N and 170 N did not show significant differences (Figure 2). The overall differences among fertilizer levels could be observed during each individual harvest.

**Figure 3:**
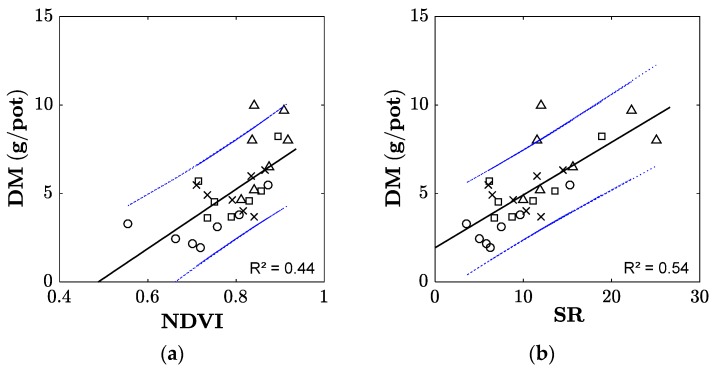
Relationship between above-ground *Lolium perenne* dry matter of the fourth harvest and vegetation indices ((**a**) NDVI; (**b**) SR). The dotted black line represents the function obtained by linear regression, and dotted blue lines show the upper and lower confidence intervals (alpha = 0.05). Different symbols represent different fertilizer levels: ∆—340 N; □—170 N, ×—85 N and ○—control.

Differences in the above-ground plant biomass were also detectable using the NDVI and the SR (Figure 3). The observed linear relationship between the vegetation index and dry matter yield was slightly stronger for SR than for NDVI. Standard errors of the predictor and intercept were respectively 2.9 g/pot and 3.7 g/pot for NDVI, and 0.05 g/pot and 0.65 g/pot for SR. Nevertheless, the vegetation index values in particular showed substantial variability—especially for the control and 340 N treatments.

### 3.3. Soil C and N Dynamics

**Figure 4:**
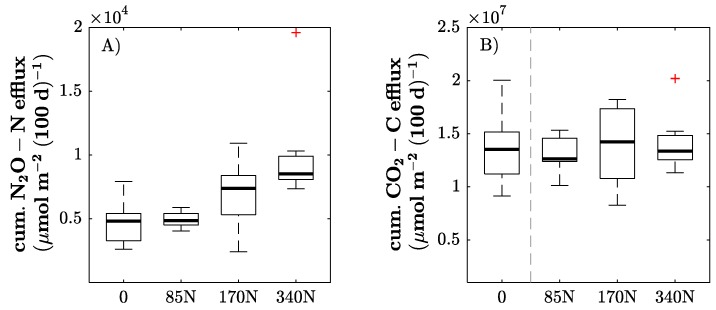
Responses of cumulative N_2_O–N efflux (**A**), and cumulative CO_2_–C efflux (**B**) to applied amounts of calcium ammonium nitrate in kg·N·ha^−1^. Horizontal box lines represent the median; boxes show the interquartile range; whiskers correspond to ±2.8 σ; and samples extending whiskers are considered outliers, using the + symbol. Different letters within a plot indicate significant differences at *p* ≤ 0.05.

**Figure 5:**
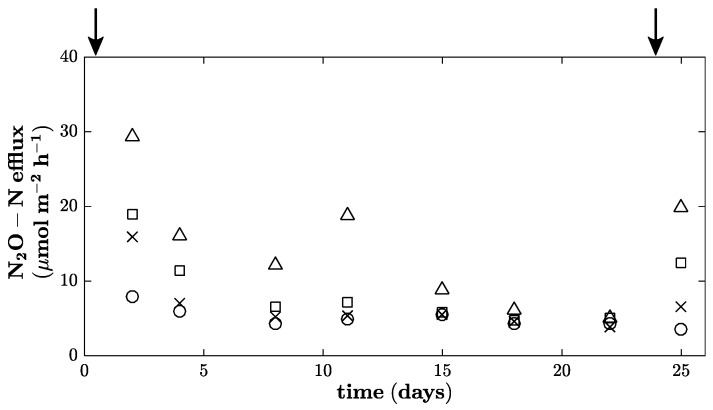
N_2_O–N efflux rates for the first 25 days of the experiment with four treatments: ∆—340 N, □—170 N, ×—85 N and ○—control (numbers indicate kg·N·ha^−1^ applied as CAN). Vertical arrows indicate fertilization and harvest.

Cumulative CO_2_ efflux showed no significant differences between fertilizer treatments (Figure 4). However, cumulative N_2_O emissions increased with the fertilizer level, even though only the largest fertilizer treatment had significantly larger emissions compared to the control and the 85 N treatment. This difference was also reflected by significant differences in inorganic N and EON at the end of the experiment (Table 2). Even though the EON values were increased in the 340 N fertilizer treatment, this was not associated with higher extractable organic C. The N_2_O emissions increased after every fertilization event (following harvests), as exemplified in Figure 5 for two harvests and the intermediate period. The figure also shows that the extent of the N_2_O increase after harvesting and fertilization was strongly related to the fertilization level. Nevertheless, MBC and MBN did not show any significant differences among fertilizer treatments. Additionally, plant biomass did not show any relationship with soil MBC, MBN or inorganic N content. Likewise, the NDVI was not related to any of the assessed soil traits.

## 4. Discussion

### 4.1. Sensing Plant Traits

It has been shown that plant traits such as fresh plant biomass, plant dry matter, plant N and chlorophyll content can be easily determined using optical sensors able to detect light reflection in the range of red to near-infrared light [7,9]. Depending on the wavelength detected by the sensors, different vegetation indices can be calculated. In our experiment, the optical sensor GreenSeeker provided the possibility to calculate the two vegetation indices NDVI and SR, which were correlated with increasing biomass caused by N fertilization levels. However, the relationship was rather weak, as indicated by moderate R^2^ values and as was also shown by others for cereals [21] and in grassland [26]. Even though the soil moisture and temperature were kept constant in a narrow range, and light conditions were expected to be relatively homogeneous due to the use of artificial light in the greenhouse, the variability between individual pots was substantial for the plant dry matter and vegetation indices. In the field, the relationship between the NDVI derived from GreenSeeker measurements and plant biomass might even be weaker, due to a stronger variability of environmental factors, such as soil moisture and temperature, spatial heterogeneity, and varying light conditions, which could affect the stability of sensor readings. Consequently, simple optical sensors might not be a suitable tool for assessing grassland biomass in the field, especially when taking into consideration that grassland is usually more diverse than the *Lolium perenne* sward we investigated. Future experiments should consider using hyperspectral scanners/sensors to allow for the calculation of further vegetation indices, which might be better suited to detect differences of plant biomass or N levels in grasslands, as shown already in other more recent studies [7,9]. Hyperspectral scanners provide information from a wider range of spectral bands, which allow the calculation of a range of different vegetation indices. These may allow for the detection of differences in plant traits of differently fertilized grassland swards, especially under field conditions, where environmental factors such as water availability affect spectral reflectance patterns of plant canopies [9]. Hyperspectral data can also be used to determine a wider range of plant traits [7], such as plant height, which is also closely correlated to sward biomass [14]. Hollberg and Schellberg [9] propose using data from hyperspectral scanners, which would enable a decision on the optimal vegetation index to be used in relation to given environmental conditions in the field, and they suggest considering a combination of different vegetation indices to optimize the prediction of plant traits in grasslands. This approach should be tested in future experiments under field conditions to elaborate on the underlying mechanisms of the relationship between the spectral reflectance and plant-soil interactions.

Even though the calculated vegetation indices showed a relationship with the plant biomass (as increased by N fertilizer levels in our experiment), as also shown in other studies [7,9], we were not able to show a relationship between the vegetation indices or plant biomass and N_2_O emissions, although the N_2_O emissions were also elevated with the increasing N fertilizer addition. Moreover, there was no relationship between the plant biomass or NDVI, and the soil microbial biomass, EON or inorganic N as products of microbial activity. Therefore, changes in the spectral reflectance of the plant canopy in relation to changing N levels cannot be expected to provide information on soil microbial properties or microbial-driven soil processes, even though these (N_2_O emissions, inorganic N and EON) are to a certain extent related to the fertilizer level as well.

Future experiments aimed at elucidating the relationship between plant above-ground biomass traits and soil properties, such as N_2_O emissions or other N fluxes in the soil, have to consider using sensors with a broader detection spectrum (e.g., hyperspectral sensors). This would allow for exploring whether a relationship between the fertilizer addition or N level, plant biomass and chlorophyll content, and N_2_O emissions can be determined indirectly. Nonetheless, overall it remains questionable whether soil microbial properties or products of their activity, such as NO_3_^−^ or N_2_O emissions, or even changes in the microbial community composition [11], can be detected reliably using reflectance patterns of plant surfaces. Especially for the latter, a mechanistic explanation is not evident. Even though a mechanistic relationship between the N fertilizer level and N_2_O emissions or chlorophyll content is established, N_2_O emissions cannot yet be predicted by measuring changes in the plant surface reflectance. Spectral data should thus be used to more reliably detect changes of plant traits in space and time, which can feed into a plant growth model in which N pathways are parametrized based on other experiments. This model would then allow the prediction of N demand by plants. Moreover, combining multiple sensors, such as hyperspectral sensors for vegetation measurements, photoacoustic trace gas detectors, and soil sensor networks for monitoring environmental conditions, may also contribute to elucidating these relationships in experimental setups.

Pot experiments in a greenhouse provide conditions where certain environmental factors can be better controlled. This allows for the evaluation of relationships between N fertilizer addition, plant biomass and N fluxes, which are more strongly masked under field conditions, where field heterogeneity and temporal variability is more pronounced. However, pot experiments can also suffer from drawbacks such as a higher soil temperature than in the field, resulting in an increase of temperature-dependent soil processes, such as C and N mineralization [27]. Moreover, strong changes in the soil moisture can occur in pot experiments, due to high transpiration rates and relatively low water availability in a small soil volume, especially when ambient temperatures temporarily increase. As a solution to the problem, soil moisture and soil temperature sensors can be coupled with irrigation systems, thereby reducing strong fluctuations in the soil temperature and soil moisture. This can reduce stress on plant and soil microorganisms, and avoid artificial measuring conditions. In addition, using sensors for continuous soil temperature and moisture detection allows for the determination of the right time points for measuring temperature- and moisture-sensitive soil processes, as shown for N_2_O emission rates in our study. Consequently, sensor networks can be useful tools for planning measurement regimes, guaranteeing comparable results collected at comparable conditions. This might be particularly useful for pot experiments under controlled conditions, but it is also useful for field experiments, where the moisture variability will be of major importance for various soil processes and plant development.

### 4.2. Soil C and N Dynamics

As expected, inorganic N fertilization increased the above-ground plant biomass and yield due to larger amounts of available N. This, however, did not result in an increase in CO_2_ emissions, which were independent of N fertilization, indicating no enhanced root or microbial activity. At the same time, increasing the N availability, as reflected by higher amounts of inorganic and extractable organic N in the 340 N treatment, resulted in higher N_2_O emission rates. There was a significantly positive, non-linear response of the N_2_O emissions to the amount of N input. This was especially evident at higher fertilizer rates. Whereas the N_2_O emission rates of 170 N remained at the same level as the control, the 340 N treatment had almost double the emission of the control. Shcherbak et al. [28] determined that the N_2_O response to applied N increased exponentially, which is confirmed by the results of our pot experiment. Nevertheless, a larger number of different fertilizer rates, covering the gaps between the existing four, would contribute to confirming the exponential response. Besides that, elevated N_2_O fluxes were measured directly after the fertilizer application, as demonstrated in other grassland studies [29,30]. As our measurements took place 1 to 2 days after the fertilizer was added, it cannot be excluded that cumulative N_2_O emissions in our experiment were underestimated, as the major peak of the N_2_O release occurred right after the N addition. Thus, the emission rates of the fertilized treatments in our experiment decreased non-linearly during the growth period, and finally reached the same level as the control. Overall, the N_2_O emission rates of the control were constant over time, and no peaks were observed immediately after the harvests, which corresponds with the observations of Glatzel and Stahr [31]. Furthermore, all soil samples of the pot experiment were adjusted to a 50% water holding capacity so that the effect of rainfall on N_2_O emissions, especially after the fertilizer application, could not be investigated. Not all events causing a major release of N_2_O fluxes over the year could be implemented in the greenhouse experiment, so that upscaling to annual release rates is not feasible. Excluded from the experiment were site-specific events, such as rainfall, frost-thaw cycles, and dry-wet cycles. During winter, Hoffmann et al. [32] detected peaks of N_2_O emission rates on the same grassland site where the soil samples were taken. Although the Lower Rhine is one of the regions with the shortest periods of frost in Germany (57 days per year; January: 14 days), the significance of these events to the N_2_O release is not negligible. Thus, our results reflect the exclusive effect of N fertilizer addition and N availability on N_2_O emissions. Ultimately, N_2_O emission rates are not only controlled by the total fertilizer N input to the system. There are also strong multi-factorial site effects [33,34,35] such as pH, soil water content, temperature, texture, soil aeration status, and carbon availability that regulate and influence N_2_O emissions obtained from chemical and microbial sources.

The MBC and MBN content of the soils in our experiment were in the range of other studies on temperate grasslands [36,37]. Even though the soil was from the same field as that used in a previous incubation experiment [6], values were smaller. Even though there is evidence for positive indirect effects of inorganic fertilizer addition on soil microbial communities, by increasing plant growth and therefore facilitating C input by plants, negative effects, such as a reduction of the pH, can also be expected after enhanced inorganic N fertilization [38]. Most of these observations, however, were derived from arable systems. In permanent grassland systems, the soil microbial biomass is clearly more negatively affected by inorganic N addition in the long-term, which is probably caused by decreasing plant species’ richness, and is only affected positively in the short-term [39]. As in a previous study using the same soil [6], microbial biomass was not affected by the N fertilizer level after the addition of inorganic fertilizer, which is probably due to the overall high microbial biomass level, which can better cope with stress [40]. Additionally, the high clay and organic matter content of the grassland soil used may have buffered possible negative effects of the inorganic fertilizer on soil organisms, such as osmotic stress or toxicity. Moreover, the sward was dominated by *Lolium perenne*, and thus negative effects of increasing the N on plant species’ richness were not existent. Consequently, no changes of the soil microbial biomass could be detected in this rather short-term experiment.

Some studies showed a close negative relationship between the EON and inorganic N or microbial biomass [41,42], where the EON was often used as a C source by the microorganisms resulting in release of inorganic N. On the other hand, EON can also be a source of N, if N is limited [43]. In contrast, the EON in our study increased along with the inorganic N when inorganic N fertilizer was added. Thus, soil microorganisms clearly did not make use of the EON as a C nor N source in our study, likely because N was available in this fertile soil and C was sufficiently provided by the plant roots as rhizodeposits. On the other hand, the slightly elevated EON levels at the highest fertilization level in our study might have reflected an increased microbial turnover, and thus consisted of microbial residues. This, however, was not reflected by significant changes in the soil respiration or microbial biomass, and overall was not the focus of our study; it therefore remains speculative.

There was no correlation between the investigated plant traits or soil N parameters (inorganic N and N_2_O emissions) and the microbial biomass. Consequently, the soil microbial biomass or microbially-driven processes cannot be determined indirectly using optical sensors and simple vegetation indices as discussed above. When aiming to model plant biomass development and N uptake in grasslands, soil microbial processes of N mineralization and N_2_O release need to be considered, especially as the microbial biomass is larger in grasslands than in arable systems. However, these possible model parameters can likely not be adjusted using remote sensing data.

## 5. Conclusions

Our results confirm that simple optical sensors and simple vegetation indices can, to a certain extent, be used to detect differences in the plant biomass of *L. perenne* dominated grasslands as affected by N fertilizer levels. Additionally, as expected, elevated N levels resulted in increased N_2_O emissions, even under the aerobic soil conditions. However, despite the relationships between the N fertilizer level and biomass on the one hand, and the N level and N_2_O emissions on the other hand, no relationship of the N_2_O to the biomass or vegetation indices existed, thus not allowing for an indirect assessment of N fluxes using simple optical sensors. As microbial biomass and mineralization patterns were not affected by the N fertilizer level in the nutrient rich soils, it can be proposed that plant–soil feedback with respect to N dynamics is probably less intensive. As a consequence, we conclude that simple optical sensors cannot be used to assess N fluxes in the soil-atmosphere continuum. Future studies have to elaborate on the possibility of assessing N_2_O emissions and other N fluxes, based on plant traits using more complex sensors and sensor networks.

## Figures and Tables

**Table 1:** Selected vegetation indices of the fertilization experiment.

Vegetation Index	Formula	Source
NDVI	$\left(R_{770\;\mathrm{nm}}-R_{650\;\mathrm{nm}})/(R_{770\;\mathrm{nm}}+R_{650\;\mathrm{nm}}\right)$	[20]
SR	$R_{770\;\mathrm{nm}}/R_{650\;\mathrm{nm}}$	[21]

**Table 2:** Microbial biomass C (MBC), microbial biomass N (MBN) and inorganic C content, as well as extractable organic carbon (EOC) and extractable organic nitrogen (EON) of the fertilizer treatments—control, 85 N, 170 N, and 340 N (numbers indicate kg·N·ha^−1^ applied as CAN)—after the last harvest (day 100). Different letters within a column indicate significant differences at *p* ≤ 0.05. Values represent mean ± SD for *n* = 7.

Treatment	MBC (µg (g^−1^ soil))	MBN (µg (g^−1^ soil))	Inorg. N (µg (g^−1^ soil))	EOC (µg (g^−1^ soil))	EON (µg (g^−1^ soil))
Control	602 ± 82 a	121 ± 19 a	8.9 ± 1.9 b	71.8 ± 6.6 a	22.8 ± 4.3 b
85 N	608 ± 83 a	124 ± 17 a	10.0 ± 1.2 ab	76.6 ± 5.8 a	25.8 ± 4.2 ab
170 N	559 ± 71 a	109 ± 19 a	9.6 ± 2.1 ab	74.0 ± 13.7 a	25.6 ± 4.7 ab
340 N	547 ± 80 a	106 ± 16 a	12.8 ± 3.1 a	74.9 ± 11.7 a	34.5 ± 9.4 a
